# System, Subsystem, Hive: Boundary Problems in Computational Theories of Consciousness

**DOI:** 10.3389/fpsyg.2016.01041

**Published:** 2016-07-27

**Authors:** Tomer Fekete, Cees van Leeuwen, Shimon Edelman

**Affiliations:** ^1^Department of Psychology, KU LeuvenLeuven, Belgium; ^2^Department of Psychology, Cornell UniversityIthaca, NY, USA

**Keywords:** consciousness, open dynamical system, brain dynamics, integrated information, representational capacity, trajectory space, systemic properties, intrinsic

## Abstract

A computational theory of consciousness should include a quantitative measure of consciousness, or MoC, that (i) would reveal to what extent a given system is conscious, (ii) would make it possible to compare not only different systems, but also the same system at different times, and (iii) would be graded, because so is consciousness. However, unless its design is properly constrained, such an MoC gives rise to what we call the *boundary problem*: an MoC that labels a system as conscious will do so for some—perhaps most—of its subsystems, as well as for irrelevantly extended systems (e.g., the original system augmented with physical appendages that contribute nothing to the properties supposedly supporting consciousness), and for aggregates of individually conscious systems (e.g., groups of people). This problem suggests that the properties that are being measured are epiphenomenal to consciousness, or else it implies a bizarre proliferation of minds. We propose that a solution to the boundary problem can be found by identifying properties that are *intrinsic* or *systemic*: properties that clearly differentiate between systems whose existence is a matter of fact, as opposed to those whose existence is a matter of interpretation (in the eye of the beholder). We argue that if a putative MoC can be shown to be systemic, this ipso facto resolves any associated boundary issues. As test cases, we analyze two recent theories of consciousness in light of our definitions: the Integrated Information Theory and the Geometric Theory of consciousness.

## Introduction

Computational theories of consciousness (CTCs; e.g., Fekete and Edelman, 2011; Oizumi et al., 2014) attempt to answer the question of how physical systems give rise to phenomenal experience by appealing to the computational structure of their dynamics. CTCs typically adopt a very abstract perspective on this problem, and hence restrict themselves to minimal assumptions about phenomenal states (and therefore about conscious states in general)^1^. Prima facie this perspective lends itself to understanding not only human consciousness and its relation to neural dynamics, but also animal consciousness, as well as the possibility of artificial consciousness.

One of the key observations regarding phenomenal experience is that it appears to be graded: not only can otherwise active brains fail to give rise to experience at all (e.g., under anesthesia), but, moreover, the richness of our experience varies greatly over time. Our growing understanding of complex systems theory suggests that this is not a coincidental feature of human consciousness, or even of neuronally based experience in general, but rather an expected one: by systematically varying the composition of a system (model) realizing certain dynamics and/or the parameters that control its activity, it is typically possible to degrade and otherwise vary the salient computational properties that the system exhibits. Consequently, as CTCs are founded on the notion that what makes for phenomenal experience is the realization of computational properties, it follows that graded experience is an all but inescapable feature of CTCs.

It is therefore unsurprising that CTCs typically imply the possibility to measure the extent to which given dynamics realize phenomenally rich content, and go as far as suggesting measures of consciousness (MoCs): real-valued functions defined on the system's dynamics, whose output is designed to vary monotonically with richness or “degree” of consciousness. This is roughly equivalent to quantifying the extent to which a model realizes the computational properties that are assumed to underlie phenomenality. Ideally, an MoC should support fine-scale and clearly interpretable comparison between different regimes under which the same system (model) can operate, as well as the comparison of systems that differ in their composition. Such an MoC whose results can be expressed in standardized units would be universal, in that it would apply to any physical system and would provide a metric to compare and rank systems—a “consciousness detector.”

The theoretical appeal of MoCs is clear: they offer a principled means of answering highly contested questions such as the extent of consciousness in living organisms (are cats conscious? are slugs? is a nervous system necessary for having experience?), rather than simply affirming our preconceived notions as to the place of experience in nature. An MoC that would apply at least to primate brains would also have a great practical significance. Consider for example intraoperative awareness, affecting about one in a thousand people under anesthesia (American Society of Anesthesiologists Task Force on Intraoperative Awareness, 2006), who unfortunately have some experience of undergoing a surgical procedure. This experience may vary from dim and fragmented to quite vivid, and can be very traumatic and lead to debilitating post-traumatic symptoms. To minimize intraoperative awareness, BIS (bispectral index) machines are often used. A BIS machine monitors EEG signals, ranking them on a scale from 0 to a 100 (denoting, respectively, EEG silence and full-fledged alertness), with levels of 40–60 supposedly indicating sufficient levels of anesthesia, that is, absence of consciousness. Thus, in effect the BIS is an MoC, albeit not a theory-driven one, but simply a result of data fitting (the exact details of BIS machines are not disclosed by their manufacturers).

Clearly, a theory-driven MoC must do better than such crude measures. Indeed, it is hard to see how a theory of consciousness could be considered complete without specifying a universal MoC. Doing so, however, is not an easy undertaking. Here we state and discuss a cluster of problems arising from the notion of an MoC. Specifically, the following are all aspects of what we refer to as the *boundary problem*:

1. An MoC indicating that a given system is conscious will do so for (at least some) subsystems of that system. We call this the *system/subsystem* problem.
2. An MoC indicating that a system is conscious will do so also for the same system augmented with some extraneous elements. We call this the *embodiment* problem.
3. An arbitrary conjunction of systems that an MoC labels as conscious would also be accounted conscious. We call this the *hive mind* problem.

The reliance on an MoC thus gives rise to a situation in which declaring a system to be conscious forces us to admit the same for a multitude of other systems, many of which are virtually identical to it.

It is important to note that this problem extends to any computational theory of experience—an MoC is, after all, nothing more than a clear articulation of the computational property that realizes graded experience. A viable CTC must thus include an explanation of what it is that makes a system different from the set of its parts, as well as what constitutes the boundary that separates it from other systems. This is by no means a contrived or purely theoretical problem: the usefulness of a CTC and of an MoC that accompanies it depends entirely on how convincing it is when applied to real-life situations ranging from anesthesia, through presence of consciousness in brain-damaged patients (e.g., locked-in states), to theoretically interesting questions regarding consciousness in split brains, computers, and alien life forms, as well as questions regarding the necessity of embodiment for consciousness.

These observations leave us with several options. We could take the aforementioned problems as an indication that an MoC is not possible. This choice effectively gives up on the possibility of developing a computational theory of consciousness—an unappealing prospect, given how pervasive and successful the computational approach is in relation to all other aspects of the mind/brain (for a review see Edelman, 2008). Alternatively, we could bite the bullet and accept the consequences, including the bizarre proliferation of minds implied by the system/subsystem and the hive mind problems. This stance not only would be lacking in theoretical parsimony, but would also be overly bold: all our intuition and all the data we have suggest that consciousness arises exclusively in single-embodied, indivisible minds.

The remaining option is to impose additional constraints on the application of MoCs. For instance, one could try to show that applying an MoC to a given substrate (putative system or set of systems) results in quantitative differences between single- and multiple-mind scenarios. A more ambitious goal would be to show that the difference between such cases is qualitative.

In the remainder of the paper, we discuss the possibilities for developing MoCs that would result in such quantitative or qualitative distinctions. In Section Model selection, The Boundary Problem, and Inter-Theory Convergence, we focus on the former, by considering whether it makes sense to employ some sort of model selection criterion, with the aim to show that the results of MoC analysis are best explained by a single mind when applied to a healthy awake person, and conversely by two or more when applied, for instance, to a split brain (if this is indeed the case). In the subsequent sections, we explore the possibility of making a qualitative distinction, by identifying a family of properties of open dynamical systems—systems of elements whose behavior is determined through mutual interaction and concurrent external influences—that single out wholes rather than parts or aggregates. To that end, in Section Implementation and the Boundary Problem we analyze the concept of implementation.

The notion of implementation—what it means for a system to implement a computation—has long been considered insufficiently constrained (Putnam, 1988), casting doubt on its value as an explanatory device in understanding the mind. At the same time, any quantitative theory in physics rests on the concept of implementation (of the laws of physics, by a given system), which implies that implementation is not conceptually problematic as such. This observation suggests that our focus should be on capturing the exact sense in which implementation is problematic insofar as experience is concerned.

To begin with, we note that any account purporting to explain experience must be intrinsic: the experience of a system cannot be a matter of interpretation by an external observer (Balduzzi and Tononi, 2008; Fekete and Edelman, 2011; Oizumi et al., 2014). Of course, for a property (or a description) to be independent of external points of view (other possible descriptions) does not necessitate that it have any first-person aspects. We contend however, that the converse must be true: that the encapsulated nature of experience can only arise through instantiation of intrinsic physical properties. Consequently we wish to spell out the constraints that must be satisfied for a CTC to be independent of an external points of view—a running theme for the rest of the discussion. To this end we discuss what it means for certain computational properties to be intrinsically realized.

Accordingly, in Section The Boundary Problem and Systemic Properties, we proceed to analyze the relationships between systems (wholes) and subsystems (parts). In particular, we offer an analysis in terms of dynamical systems of the situations in which “the whole is greater than the sum of its parts” (Aristotle, Metaphysics H) and suggest that a better formulation for the case of MoCs would be “the whole is different from the sum of its parts.” This leads to the notion of *systemic properties*—properties that make it possible to state the conditions under which a system, over and above the set of its parts, exists intrinsically and is not merely as a matter of attribution. We show how an appeal to such properties can make a CTC immune to the boundary problem and consider whether or not they could serve as a naturalistic way of modeling the unity of consciousness.

In Sections Tononi's IIT: Is Integrated Conceptual Information a Systemic Property? and The Geometric Theory Revisited, we apply these conceptual tools to two computational theories of consciousness: the Integrated Information Theory of Tononi (Oizumi et al., 2014) and the Geometric Theory of Fekete and Edelman (2011, 2012). Doing so reveals some of the strengths and weaknesses of the two theories, not only in dealing with boundary issues, but also, just as importantly, in how intrinsic they are in the above sense, and how they relate to the notion of the unity of consciousness^2^.

## Model selection, the boundary problem, and inter-theory convergence

One of the critical determinants of the success of mathematical models of physical phenomena is the numerical correspondence between measured and predicted quantities. All things being equal, if one model is more accurate than another, we consider it to be closer to “the truth.” In technical terms, such models strike an optimal balance (according to some predefined criterion) between goodness of fit (how well the model explains existing data) and predictive ability. The process of establishing which of several models is preferable in this sense is referred to as model selection. Unfortunately, considerations of goodness of fit/predictive ability (for the conjunction of which we will use the shorthand *adequate fit* in what follows) do not appear particularly informative in the computational modeling of consciousness: we can imagine a highly accurate model of brain dynamics which nevertheless offers no insight into the consciousness (or the lack thereof) associated with that dynamics.

However, this is not to say that a CTC is *exempt* from adequate fit requirements pertaining to the underlying dynamics: after all, it is the dynamics of a system that gives rise to experience. It is therefore important to recognize that an MoC is an additional piece of explanatory machinery, which complements a model of the pertinent (say, brain) dynamics. In particular, a successful MoC could help us evaluate the specifics (such as the equations or the range of parameters) of a model of brain dynamics, so as to focus on a particular, perhaps optimal, level of detail (e.g., neuron vs. membrane compartment or Brodmann area). Alternatively, it could help establish that only a multiscale approach will do, a notion that we revisit below.

Let us try to understand what adequate fit could mean when applied to an MoC. Insofar as the fit between model and data is concerned, we must recognize several types of *convergence*. First, there is inter-model convergence, which quantifies how well the model in question performs in accounting for the phenomena of interest (as compared to other possible models and in general). Second, there is intra-model convergence: horizontal, which involves fitting the parameters of a model such that it best explains data, and, less obviously, vertical, which involves choosing the composition of the model. Regarding the latter, suppose that we have an agreed-upon framework for modeling a phenomenon (e.g., consciousness) and suitable methods for fitting/discovering parameters; this still leaves open the question of whether or not the model encompasses the optimal (in terms of goodness of fit/predictive ability) or correct (in some other sense) complement of constituents, from among the set of all potentially relevant ones. This is the boundary problem introduced above.

Within vertical convergence a further distinction should be made, which gives rise to distinct aspects of the boundary problem, between upward and downward convergence. Upward convergence is what happens when achieving a more adequate fit requires adding elements to the putative system that is being singled out. Thus, we can ask whether or not an embodied brain is better suited for modeling consciousness than a disembodied one (as in the case of a brain in a vat), as well as whether or not a hive mind is more conscious than a single-brain one. Conversely, downward convergence is what happens when elements must be left out of the description of the system to achieve a more adequate fit, as in the system/subsystem problem.

What we need, then, is a mechanism for exploring the vertical convergence of an MoC. Unfortunately, best fit between a mind and its model is not quite practical as a criterion, because a mind cannot be accessed directly “from the outside.” For example, even if we had an MoC that predicts the level of awareness under anesthesia, its evaluation would still have to rely on querying the subject in the awake state. Apart from the usual problems of noise stemming from the questionnaire approach being necessarily indirect, such a measurement would be temporally displaced relative to the state that it is intended to assess. Furthermore, it is hard to see how it could generate data regarding the proper level (if any) for modeling brain dynamics, the contribution of a particular part of the brain such as the cerebellum or the basal ganglia (say) to consciousness, or the emergence of two minds in a split brain.

**Figure 1:**
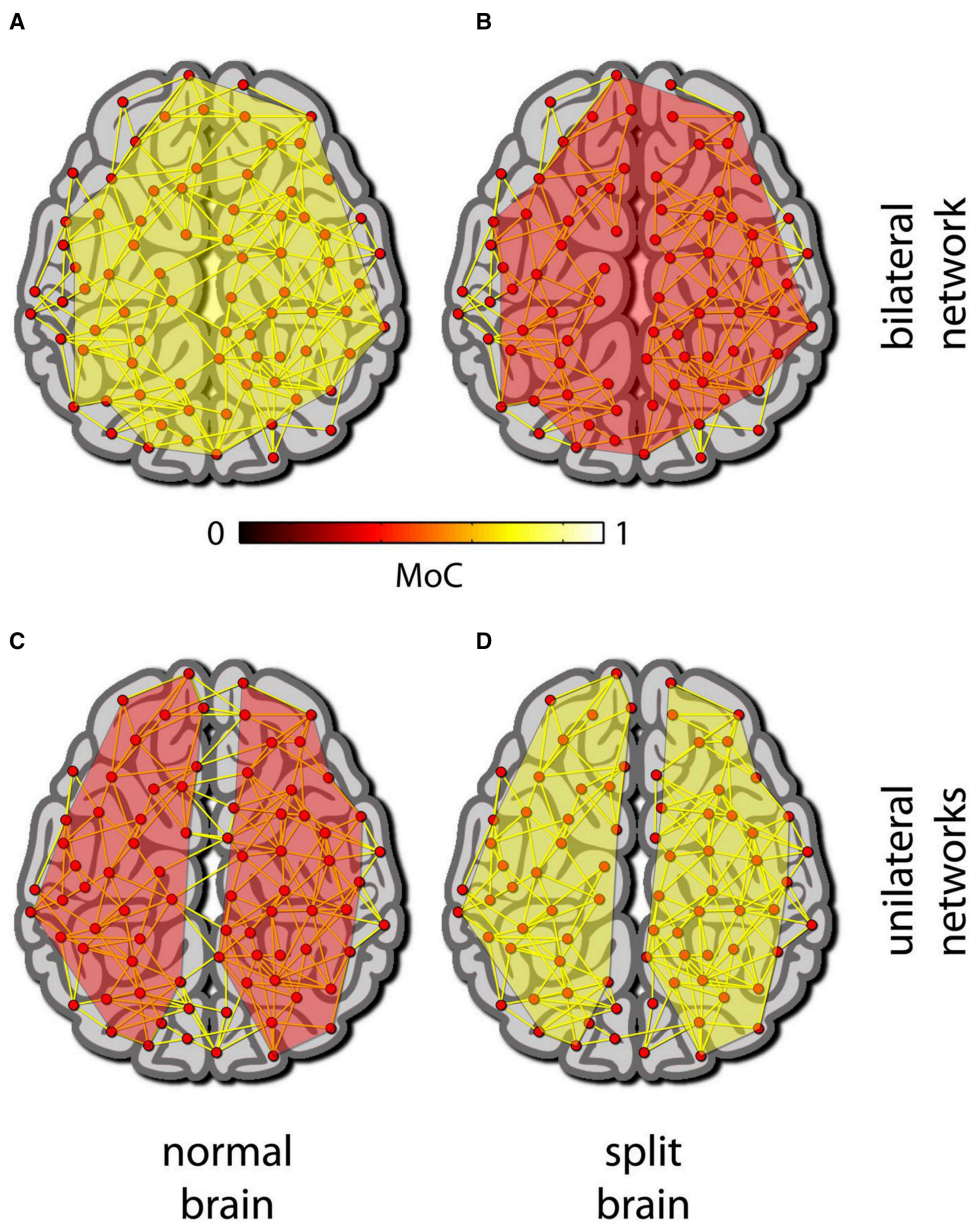
**Hot spot analysis (HsA)**. In HsA, an MoC is applied to different subsets of an array of measurements/elements in model. **(A–D)** A depiction of a normal brain (left) and a split brain (right) modeled as a set of elements and their connections. **(A)** For the normal brain the MoC gives a maximal value for a collection of elements spanning most of the brain. **(B)** In a split brain the same set of elements as in (A) gives a much lower, and non-maximal reading. **(C)** For the normal brain the maximal MoC reading in each hemisphere taken on its own is smaller than the maximal reading obtained from the whole brain network. **(D)** The opposite of (C) is true for the split brain: the maximal MoC readings are for two disparate networks each spanning most of a single hemisphere.

Still, if we do have an MoC that fits these limited data well and is meaningful from a theoretical point of view (e.g., according to IIT), using it to search for “hotspots” might help us resolve some of the above boundary problems. To do so, the MoC should be applied to the power set of all the elements of the putative system (e.g., physiological measurements derived from the brain and the body), with the exception of subsets for which the MoC is not defined (such as singletons in the case of a relational ensemble-based MoC). Thus, for example, if the MoC consistently yields higher values across subjects when applied jointly to cortical and cerebellar measurements (compared to the cortical measurement alone), then there would be a good reason to claim that the cerebellum contributes to consciousness. Similarly, if hotspot analysis of a split brain indicates a bifurcation, it would support the notion of two emergent minds whereas a single hotspot would support the converse (see Figure 1).

However, aside from being limited as already mentioned, hotspot analysis is clearly *ad hoc*. In particular, given that direct empirical verification of an MoC is inherently problematic, we would understandably be wary of trusting such analyses in scenarios that go beyond what we are familiar and comfortable with. Instead, we propose to raise the stakes, by seeking to identify “dynamical” properties—by which we mean properties of a set of equations describing a collection of elements and their interactions—that characterize “entire” systems but not their parts. To do so, we first discuss what it means for a system to implement a set of equations, that is, to realize a certain dynamics.

## Implementation and the boundary problem

We shall now offer several observations regarding the notion of implementation: a physical system realizing a formal structure through its dynamics, that is, the causal (endogenous and/or environmental) factors that steer it. To take on the boundary problem inherent in the relationship between a system and its subsystems, we need to understand the interaction between their respective dynamics. We propose to do so by stating what it means for a subsystem to operate “on its own.” Roughly, this amounts to whether or not a subsystem implements a dynamics that is different (in a sense that we shall define) when considered separately from the broader context of the rest of the elements that comprise the entire system.

In the following discussion of implementation, we adopt the notion of minimal computationalism (Chalmers, 2011). Accordingly, where possible we avoid specifying whether the implementation in question is discrete (digital) or continuous (analog), as the two theories to be discussed later as test cases differ in this regard. Because we are interested in the modeling of physical systems that change over time according to a lawful relation, we use the term dynamical system. Simply put, a physical substrate is said to implement a dynamical system specified by a formal model if (i) there is a bijective mapping between the elements of the model and the elements of the putative physical system; (ii) there is a bijective mapping between the states of each formal element and those of its physical counterpart; and (iii) given these mappings, the transition rules in both domains are isomorphic (which is to say that the causal structure of the physical substrate exactly corresponds to the transition rules of the abstract model, including all the necessary counterfactual stipulations established through measurement)^3^.

We are interested in systems that can be, in constant interaction with their surroundings, given that normally awake organisms, our paradigmatic example of consciousness, are. Such systems are referred to as open dynamical systems, and accordingly we need to modify the above definition by separating the state of the system at a point in time into several components: inputs, internal state, and parameters, where inputs correspond to concurrent streams of external perturbations to the system^4^, and parameters are aspects of the system or of its immediate surroundings that express themselves in the transition rules. In accordance with common terminology, we refer to initial conditions as the parameters and internal state at the start of the period of time during which the system is being analyzed.

The question whether or not a system implements a given dynamics can only be answered if ***all*** the possible behaviors of the system are considered (this naturally includes all possible inputs and initial states). Another way of stating this observation is by appealing to the concept of the space of trajectories. If the instantaneous state of a system is thought of as a vector, a trajectory of the system is a sequence of such vectors ordered in time^5^. The trajectory space is then the space of all possible trajectories (behaviors) of the system. To state the obvious, not only does an implementation uniquely determine a trajectory space, but two systems that give rise to two different^6^ trajectory spaces necessarily implement different dynamics. Given that we are interested in models of experience, we have in mind spaces comprising trajectories of definite duration (e.g., 100 ms, 1 s, and so on), or several such durations where multi-scale analysis is considered.

It seems intuitively clear enough a statement, that implementation can only be fully understood and evaluated in light of all possible combinations of input sequences and initial internal states. Yet, there is more than one way to construe the phrase “all possible” here. For instance, one may opt for logical possibility, which, assuming that inputs and initial conditions are encoded as arrays of numbers, implies varying their values combinatorially and exhaustively. This, however, may not be viable, due to physical limitations that single out *admissible* initial conditions: elements can only take bounded values (typically nonzero values and a maximal value bound), and there often are mutual dependencies among elements, ruling out various logically possible conjunctions of element states. Similar considerations restrict the range of admissible inputs and parameter values. Furthermore, in certain scenarios the statistics of input domains may be crucial to the computations realized by the dynamics^7^. Finally, certain required environmental variables are often considered to be merely enabling and theoretically uninteresting, and are hence not modeled explicitly.

Any theoretical account of implementation that overlooks the above factors is partial at best and quite possibly severely flawed^8^. It is especially important to enforce such constraints when evaluating an MoC defined on trajectory spaces. If leaving them out leads to a space that substantially differs in its structure, applying the MoC to it would still yield data, but those data would be meaningless or at least misleading. We will therefore refer to the overall set of constraints on initial conditions and on the implementation environment as the *normal conditions* for implementing a computational structure.

### Brains, vats, and normal conditions

To illustrate the notion of normal conditions, let us consider a (somewhat) concrete example: the embodiment problem as exemplified in brain in a vat (BiV) scenarios. BiV thought experiments typically involve providing the brain with a simulated sensorimotor environment that is seamlessly interpreted by it as being immersed in the real world. However, whether or not achieving sufficient verisimilitude in simulating the environment is feasible, to examine whether the embodiment thesis is true, it is necessary to focus on the precise ways in which the body affects what brains do, that is their dynamics. To that end let us consider the normal conditions that apply.

An embodied brain can operate in many dynamical regimes (and thus states of consciousness), some of which are maintained in near absence of external stimulation such as in certain sleep stages. If it proves possible to induce similar ongoing (spontaneous) dynamics in the absence of stimulation within an artificial setting, there would be a reason to believe that the BiV scenario is plausible. Ipso facto, this would establish that the body simply provides the brain with (part of the) normal conditions for implementing its dynamics. However, the fact that brain tissue in a vat exhibits very impoverished ongoing dynamics suggests that providing normal conditions goes beyond controlling the concentration of a few important types of molecules in the vat. Indeed, if it turns out that to uphold the dynamics it is necessary to maintain a dynamic biochemical environment that is coupled to the dynamics of the brain tissue, this would amount to putting in place an artificial construct mimicking a body. If this is the case, the embodiment thesis would be vindicated, demonstrating that bodily states do more than provide normal conditions for the dynamics of the brain. Rather this would indicate, that bodily processes must be encoded in the equations of any sufficiently elaborate model of the dynamics giving rise to consciousness.

### Normal conditions and hive minds

Our next example focuses on the constraints associated with admissible inputs under normal conditions, as they apply to the problem of hive minds. Approaching the problem from this vantage enables us to tease apart the circumstantial—as indicated by behavior, or other external, and therefore extrinsic factors—from intrinsic determinates. Let us consider a collection of bees and assume that under normal conditions the pertinent dynamical system modeling bee consciousness (such as it is) is some “neural network” model of the bee's brain. The question is then, what would be the mark of an emergent brain or lack thereof as determined exclusively by what the respective brains actually do—that is intrinsically.

One possibility is that, whether or not other bees are present, the bee's brain undergoes the same dynamics. To specify which computational structure it implements, we must consider all possible input sequences (streams) paired with the resulting (coincident) trajectories. Because the possibility of interacting with the rest of the swarm is thereby tacitly implied, the putative hive mind is, from an explanatory standpoint, superfluous, regardless of the swarm's actual presence.

Prima facie support for this scenario is found through invoking the possibility of dreaming or hallucination. In humans, any experience that is felt to be due to an external stimulus could in principle be due to a dream or a hallucination. This suggests that similar trajectories in our brain dynamics could be brought about by quite different factors: the “real thing” (an actual input) or a hallucination. This point applies, mutatis mutandis, to the case of a putative hive mind in a bee swarm.

Alternatively, if it is shown that the presence of other bees leads to an implementation of a different dynamics—i.e., that their presence is encoded in the equations governing each and every bee's dynamics, or, alternatively, that it leads to a phase transition in the dynamics—it would be another matter. An immediate implication would be that the associated trajectory spaces would differ in their structure, suggesting that this is a necessary condition for the emergence of a hive mind.

Similar considerations may point toward a way of resolving boundary problems in general. For example, a close consideration of the properties of trajectory spaces that are associated with consciousness could make it apparent what a litmus test should be for settling the question of the number or unity of phenomenal minds realized by a physical substrate. In the next section, we formalize this line of thinking by introducing the notion of systemic properties, which will enable us to revisit questions such as the emergence of hive minds.

## The boundary problem and systemic properties

When are parts and wholes (systems) not simply arbitrary categories imposed by an external observer, but rather intrinsic attributes of their own dynamics?^9^ A system (whole) should be qualitatively different from the parts it comprises. These parts, in turn, should be both distinct and in some sense independent from the whole (to be considered as parts) and at the same time should ineluctably “belong” to the whole. Accordingly, we propose to analyze wholeness, in the context of the present discussion, relative to a formal property, to be defined below, which (i) would make the unity of the whole explicit and (ii) would render it impossible for a part to pose as a whole.

To make this approach work in the framework of dynamical system analysis, we must first acknowledge what is tacitly implied: that it should be possible to identify, at each moment in time, a set of distinct units (elements). In this sense, we begin with a (putative) whole (system) and its parts (the elements that are the building blocks of the model). In this setting, and under some dynamics (paradigmatically, a set of equations), what would it mean for the putative whole (system) to be one? Ex hypothesi, it would mean that, with respect to the relevant property, a collection of elements that forms a whole (system) admits no parts (this of course does not necessarily carry over to other functional properties of the elements in question). Similarly, under this property a whole cannot be a part of some greater whole: if it were, the latter would admit a part, contradicting the initial assumption.

We require that the dynamical property used to distinguish parts from wholes mesh naturally with the machinery of an MoC. We proceed by initially focusing on formal properties of models, then moving on to discuss the implications of the notion of implementation under normal conditions.

In what follows, dynamics is represented by vectors holding the instantaneous states of a collection of elements and by rules (equations), parameterized by a set of parameters Θ^10^, that dictate how instantaneous states change over time^11^. We explore scenarios in which dynamics are either expanded or contracted, through addition or subtraction of elements to the model. The most straightforward first step is to start with a complete model encompassing all the elements under consideration, and work our way down from it, to contracted models (and dynamics) resulting from simply leaving out subsets of the elements, as well as the terms pertaining to these elements in the equations.

### Systemic properties

Let Ŝ denote the complete system specifying a dynamics: the elements, parameters, and equations. A system *S* would simply be the model specified by choosing a subset of elements *S* ⊆ Ŝ. By subsystem we will refer to any collection of elements *s* that is not *S* itself, i.e., *s* ⊂ *S*. We refer to a choice (*S*, θ), as a ***realization*** of the dynamics. A realization is a complete specification of a model—typically a set of equations, one for each element in *S*—down to the numeric values of the parameters, denoted by θ, and just as importantly the normal conditions associated with its implementation. We refer to the process of reducing the scope of a realization to that of a subsystem, by removing from the model subsets of elements as well as any terms in the equations of the full dynamics in which they appear, as ***model contraction***.

By ***dynamic property*** φ(*S*, θ) we mean a (theoretically motivated) property of the dynamics, formally stated. This can be simply a computable real-valued (or real vector-valued) function that should be sensitive to the choice of *S* (as it can lead to differing dynamics) as well as to the parameters of the model (as different parameters can also lead to qualitatively differing dynamics).

Note that at times it might be desirable to express a dynamics in terms of trajectory spaces. As this is an equivalent representation^12^, there is nothing lost by it, and it seems that it is much more natural to express some of our intuitions regarding the nature and structure of experience in terms of the structure of trajectory spaces (representational spaces). For example a dynamics could be characterized through utilizing functions defined on vector spaces, that is, functions that match a given space (in its entirety) with a scalar (or vector)—such as its dimensionality and degree of intrinsic clustering, which are thought to be crucial for establishing representational capacity. It is this sense of dynamical property that we will explore when discussing the Geometric Theory of consciousness.

Alternatively in other cases it might be desirable to characterize a dynamics through the structure of trajectories themselves. For example consider a function *f* defined on trajectories (e.g., sequences of instantaneous states of length *M*; say an MoC), matching each with a real number: *f* :*R*^*N*×*M*^ → ℝ, where *N* is the number of elements a system comprises. If for every trajectory χ under a realization (*S*, θ), *f*(χ) > 0 this suffices for establishing the dynamic property “positive under *f*.” It is this notion of dynamic property that we will explore in the section on Integrated Information Theory.

A ***proper part*** of a system under the property φ is a subsystem *s* of *S* such that $s\;{+}_{\varphi }\bar{s}\;\neq_{\varphi }S$, where +_φ_ and =_φ_ denote the notions of addition and equivalence as they pertain to φ. In the simplest scenario, if φ matches a realization with a scalar, this could stand for $\varphi \left(s,\theta \right)+\varphi \left(\bar{s},\theta \right)\neq \varphi \left(S,\theta \right)$. Another alternative, suitable if φ is defined on trajectory spaces, is $\varphi \left(s\;{+}_{i}\;\bar{s},\theta \right)\neq \varphi \left(S,\theta \right)$ where *i* is the inclusion map^13^. In this scenario φ is evaluated on full trajectories produced by the two reduced dynamics running simultaneously. Roughly speaking, the above inequality implies that *s* exhibits different dynamics when operating “on its own” as compared to operating within the putative system, and φ, at least at this instant, is not indifferent to the greater context of the building blocks of the given dynamics.

A ***normal cut*** of a system *S* is a bipartition of the elements in *S*, that is any set $\left\{s,\bar{s}\right\}$ such that $s,\bar{s}\neq \emptyset$.

A property φ is ***conjoint*** if every part of *S* is a proper part.

Conjoint properties are further classified as:

- ***Subadditive:*** if for every normal cut $s{+}_{\varphi }\bar{s}\;{>}_{\varphi }S$
- ***Superadditive:*** if for every normal cut $s{+}_{\varphi }\;\bar{s}\;{<}_{\varphi }S$
- ***Alloaditive:*** a conjoint property that is neither subadditive, nor superadditive is alloadditive.

A conjoint property is ***systemic*** for *S* if *S* is not a proper part of some greater system under this property. With this definition in mind, we can say that the member of any subset of units that contribute to realizing a systemic property “cares” about each and every other unit or collection of units within the system. For an example illustrating this definition see Appendix 2 in Supplementary Material.

Let us see how a theory of consciousness could be free of boundary problems in light of the above definitions. Consider first the system/subsystem problem. If the property by virtue of which a system in question is conscious is conjoint in the above sense, under this property all its conceivable subsystems or parts—e.g., where brains are concerned, all its significantly smaller anatomical subdivisions, from lobes and areas down to the sub-cellular machinery comprising brain cells—are proper parts^14^. This means that while each and every subsystem (subset of units) actually affects the property as realized by the whole, the subsystems cannot be said to do so independently: if they did, the exact same value of φ would obtain, regardless of whether the parts are “coupled” to the rest of the system. Conversely, in the exact sense defined by φ, a subsystem that is not a proper part is effectively decoupled from the rest of the elements at hand. Of course, the exact notion of coupling depends on the model in question.

Consider now the embodiment problem. If φ is systemic, at some point along the process of expanding the dynamics, the elements we are modeling become maximally interdependent. At that point, adding more elements to the system singled out by φ will not alter its value. This can happen if elements of a different kind are added, or if similar elements are added such that they interact with the rest of the elements in a different manner (e.g., though a different connectivity pattern). For example, if brains do not critically depend on the body for giving rise to consciousness, and φ were a straightforward MoC, then adding the body (or any part of it) to the model would leave φ within the brain unchanged.

Finally, with regard to hive minds, we note that if (embodied) brains give rise to consciousness through a systemic property φ, then they cannot be realizing a larger collective mind, because to do so they would have to be proper parts of that collective, in which case the unity achieved by their dynamics would be virtual and hence ipso facto could not be the realizer of each and every constituent mind.

If indeed systemic properties are an essential part of the explanatory machinery in computational theories of consciousness, can they be said to be intrinsic, as any viable CTC must be? Let us rehearse what it means for a systemic property to be intrinsic. First, it must be observer-independent, as consciousness is. However, as a systemic property is a dynamical property, defined with respect to a specific dynamics, it simply inherits whatever intrinsic credentials the dynamics possesses. Second, the property in question should a fortiori meet any requirements imposed on extrinsic (observational or attributional) properties. In this regard, systemic properties fare particularly well, in that they suggest how (at least some aspects) of a model can be established as being a “matter of fact.”

If we consider this last point in the context of experience, an intriguing possibility presents itself. The premise behind all computational approaches to modeling consciousness is that the formal structure of dynamics not only determines whether or not experience is realized, but is in fact (formally) equivalent to the formal properties of any resulting phenomenal experience. Given that systemic properties establish the fact of the matter in terms of the unity (or the lack thereof) achieved though dynamic properties, and given that these can be naturally expressed in terms of the associated trajectory spaces, we can tentatively offer them as (perhaps part of) the formal correspondence that makes for the unity of experience.

### Inputs and normal conditions in model contraction

Recall that according to our definition, systemic properties are contingent on the notion of model contraction. While our notion of model contraction is formally sound, its applicability to real life scenarios is not without difficulties. First, explicit care is necessary in demarcating what are to be considered inputs, elements, and normal conditions, as will be seen in the first example below. Second, just as importantly, assessing whether a dynamic property is systemic requires partitioning the putative system into normal cuts. However, when contracting the dynamics onto each side of the partition, it is crucial to maintain normal conditions: by definition, a violation of normal conditions leads to the implementation of a different dynamics. Depending on the MoC, this could give rise to different MoC values on each side of the partition, as compared to the intact system, leading to the erroneous conclusion that they are proper parts of the system. This point will be stressed in the second example below.

Our first example here revisits the question of hive minds. Given a putative MoC, to determine whether or not a collective mind can emerge when bees swarm—that is, to establish whether or not its relevant properties are systemic at the swarm level—it is necessary to establish whether or not MoC values taken for an entire swarm are invariant under normal cuts. To do so, the dynamics needs to be evaluated while interactions between the two parts of a cut are suppressed. However, as noted above, the notion of interaction is theory/model-dependent, which suggests that some very different approaches to this question may have to be explored. To illustrate this point, let us consider two of the possible theoretical approaches under which interactions among bees can be formulated: 1) The interactions are explicitly encoded in the equations governing each and every (embodied) bee brain; 2) The presence of other bees, as signaled by sound, sight, smell, and so forth, are treated as inputs.

Of course, these approaches are not mutually exclusive. For example, imagine that the “hive mode” could be dynamically gated via specific signals such as pheromones, a process that would be represented in explicit (interaction) terms in each and every set of equations describing the dynamics of a single bee brain, whereas other aspects of the swarm's presence would be treated as inputs. The crucial question is whether or not the model includes explicit interaction terms. If it does, normal cuts imply comparing the dynamics of (say) a single bee brain, with and without deactivating the mechanisms encoded as interaction terms in the model. In the present example, this would mean deactivating the pheromone receptors and glands of the single bee, then letting it loose in the hive with the swarm and the queen present^15^. Alternatively (with no explicit interaction terms), in the “bee as input” scenario, a normal cut could be obtained by removing the other bees. To keep the comparison of the dynamics before and after taking the cut fair, the removed bees should be replaced with holograms of recorded dances on the removed side of the partition in the same conditions. If that were technically possible, it would make it possible to assess the dynamics while keeping its interactive (reactive/dynamic) aspect suppressed (this aspect is presumably what underlies the swarm's hive mind, by instantiating a communal computation). We will revisit the question of the (im)possibility of hive minds in light of two suggested frameworks for measuring consciousness that we shall discuss in the following sections.

In our second example, we focus on the difficulties with model contraction that stem from the normal conditions for implementation. Let us return for a moment to the question of whether or not the cerebellum (say) contributes to the experience that a brain helps to realize. To address this question, we must be able to specify what it means to implement the contracted brain dynamics that exclude the cerebellum. It may be possible to assess the dynamics of a brain with the cerebellum (or some other part) temporarily and reversibly deactivated; if this proves to have no impact on the pertinent dynamical property, it would settle the above question. However, for other parts of the brain, such as the hypothalamus, the brain stem, or the basal forebrain, deactivation would lead to a catastrophe. Does this indicate that the brain stem (say) takes part in realizing experience, or simply that deactivation is not an appropriate means for implementing contraction?

To complicate things further, it is conceivable that deactivating a brain structure would lead to noticeable effects simply by effectively changing the normal conditions for networks in its vicinity, e.g., by changing the electrochemical environment of these structures, leading to altered intrinsic function of neuronal networks. In the same vein, we have shown elsewhere (Fekete and Edelman, 2012) that relegating salient aspects of brain physiology to the background role of normal conditions by considering them as housekeeping can lead to a radically different perspective on neural replacement scenarios. The upshot of these considerations is that special care is needed with regard to the assumptions associated with establishing normal conditions. We will revisit this issue in the next section, where we discuss Tononi's Integrated Information Theory (IIT) while considering its take on contracted dynamics.

## Tononi's IIT: Is integrated conceptual information a systemic property?

Tononi's Integrated Information Theory (Tononi, 2004, 2011; Balduzzi and Tononi, 2008; Oizumi et al., 2014) is the only theory of consciousness that we are aware of that offers an explicit measure of level of consciousness, expressed in absolute units that apply to all possible discrete element information network architectures. In the following discussion of IIT, we will focus more on the conceptual framework afforded by IIT, and less on the technical details involved in estimating the amount of integrated conceptual information that a system gives rise to at a given point in time. According to IIT, a (putative) system comprises a set of elements that can assume discrete states, as well as a specification of the transition rules among such states (as determined by interaction among elements). While IIT aims to describe sets of elements that influence each other's states by direct physical causal interactions (modeled after synapses or nerve tracts), it can be applied to any set of elements taking discrete states. The goal of IIT is to distinguish between intrinsic systemhood and systems that are merely a matter of attribution.

The IIT is based on the notion of integrated information (II). For any (sub)set of elements in a given state (of activation), referred to as a ***mechanism***, the amount of II it gives rise to is evaluated relative to the possible past and future states that could have caused/would likely have resulted from the current state. II relative to possible past (future) states is defined as the distance between (a) the probability distribution of previous (next) states that could have caused (resulted from) the current state and (b) the probability distribution derived from partitioning the system into parts such that the difference between the distributions is minimized (referred to as the minimum information partition)^16^. Finally II is defined to be the minimum of the past and future II. Thus, II measures the extent to which a current state is informative relative to its causes and effects, when the entire mechanism (set of causal interactions and an activation state) is considered, over and above that afforded by its parts (sub-mechanisms).

IIT recognizes the distinction between intrinsic and extrinsic perspectives: the amount of II will differ if the mechanism is considered as part of different subsets of elements. IIT postulates that only the maximal II exists, that is, is intrinsic, and suggests that to find it II should be evaluated relative to all possible partial inputs and outputs (past and future states). Any mechanism that gives rise to nonzero maximal II is then referred to as a ***concept***, as it singles out a partial set of input and output units (partial states) that have the most informative (i.e., strongly causal) ties to the mechanism. This relation captures some of the intuitions we associate with concepts, namely, that certain definite features of recent/momentary experience imply a conceptual distinction, and a concept in turn implies certain likely aspects of the subsequent experience. The reduced set of partial input and output states identified in the process is referred to as ***core cause*** and ***core effect repertoires***, respectively.

We can see that within a collection of units in a given state, several such concepts can be realized at the same time and form complicated (e.g., compositional and nested) structures. A constellation of concepts, and through that the current state of a (putative) system, can be evaluated for their integrated conceptual information (ICI), which generalizes the notion of II to the system level. ICI is computed by taking the weighted sum of the difference between the II of the constituents of the constellation of concepts contained within a set of units and the maximal II given across all unidirectional bi-partitions of the collection of units^17^, quantifying the conceptual information that emerges from the whole over and above the conceptual information that inheres in the parts of the system. The II of each concept is weighted by the distance between the core cause and effect repertoires before and after the partitions. The ICI for any given subset of elements is denoted by Φ.

IIT holds that Φ directly measures, and is in fact identical to, the amount of realized experience by a putative system (if any) in a given activation state, with one fundamental qualification. While Φ can be applied in many ways that are observer-dependent (extrinsic), only those realizations of Φ exist (that is, are intrinsic) that are maximal in the sense (i) that a different system would result if one varies the spatial grain of the description (e.g., neurons vs. cortical columns) or its temporal grain (e.g., ms vs. s) and (ii) that Φ can be applied to any collection of objects. Therefore, consciousness in a collection of elements S in an activation state exists if and only if (1) Φ > 0 for S; (2) for every subsystem of S (s⊂S), Φ is smaller; (3) S is not contained in any larger system S' for which Φ is greater. A system S satisfying these conditions is referred to as a *complex* and the constellation of concepts it forms is referred to as a *quale*. This is the *exclusion* principle: a complex is said to exclude any other systems giving rise to nonzero ICI that it includes or is included in.

Seeing that Φ is a (theoretically) computable real function, it would seem to fall under our definition of a superadditive systemic property: Φ is established relative to normal cuts, therefore by construction it is greater than the ICI of produced by its subsystems on their own, and if it is not contained by any system with larger Φ, it is not a proper part of any larger system^18^. Accordingly, IIT purports to solve both the system/subsystem and hive mind problems (Tononi and Koch, 2015): Aggregates of minds cannot fuse—while they do give rise to an irreducible causal structure, and hence to nonzero (and non-trivial) ICI, it is nevertheless smaller than the ICI produced by each brain on its own and therefore is excluded by them. Similarly, by definition a brain giving rise to a complex excludes its sub-mechanisms from being conscious.

However, until proven otherwise, ICI cannot be regarded as a systemic property, because, on the face of it, it does not satisfy our definition of a dynamic property: it is a property of a system in an activation state (a complex of mechanisms in a state), and not of a system under a realization of a dynamics. This of course raises the question why impose this requirement in the first place, and therefore whether it makes sense to relax this constraint. First, we note that it would be strange indeed if a systemic property were not always true of a(n intact) system. That said, we would like to carefully examine the ramifications of having an activation state dependent definition for ICI. We will argue that in order to capture some of the most basic properties of consciousness as found in humans, a future fully developed IIT will have to all but prove that exclusion is dynamic (and hence systemic—in the sense specified in the example “positive under *f*” in Section Systemic Properties) in the sense we introduced. The logic behind our arguments applies to any activity state-based definition of systemic properties.

### The spatiotemporal grain of experience

As noted by IIT, our experience indeed has typical spatiotemporal grain (at least for a given state of consciousness). This is part of IIT's explanatory appeal: ICI computation can single out the pertinent grain while seeking maximal ICI across spatial and temporal scales, as well as collections of elements. However, for these claims to be effective, the maxima of ICI should be unique: that is, the same maximal value of ICI cannot result from considering a system at highly disparate grain levels. Similarly, it needs to be shown, at least for systems like the mammalian brain, that typical grain is invariant. Perhaps, it is sufficient to show that this is the case within a given state of consciousness, assuming that these can be naturally parameterized for the model in question. But plausibly, given that states of consciousness seem to form a low dimensional manifold (Hobson et al., 2000), with regions which at the least seem to be graded in their properties (wakefulness would be a region which would vary in terms of properties such as the richness of experience and other qualitative differences—e.g., alertness and drowsiness), we would require that such gradients be shown to result naturally through ICI computations for a given architecture.

### The number of minds realized by a system

There is overwhelming evidence to the effect that intact (normal) brains give rise to a single mind during conscious states. Therefore, it needs to be shown that an architecture like the mammalian brain gives rise to a single maximal complex that is substantially larger than any other ICI bubbles (that IIT is willing to accept as) existing alongside it, not per activity state, but across the board. Alternatively, if it were shown that under certain circumstances this is not the case, this would amount to a critical testable prediction.

### Conceptual coherence

Under IIT a complex in a state of activation gives rise to a conceptual structure. However, IIT does not provide us with tools or even an intuition as to the structural properties of such concepts given a particular system. For example, we would expect that at least within a given state of consciousness it could be shown that the resulting conceptual structures are of similar complexity (or have some typical structure), and moreover we would want to see that for possible successive states of activation such structures are, if not identical, at least highly similar in the overwhelming majority of cases. Otherwise it is hard to see how consistent behavior could be explained, especially behavior that can only be performed consciously. In fact, it is plausible that more stringent constraints are called for, given that thought and behavior seem consistent over longer time scales, and that conscious and unconscious mental processing typically mesh together seamlessly.

### The locus of a complex within a brain

At its present state of development, IIT cannot point to where one could expect to find the concurrent complex, say in a human brain-like model in a “conscious” regime. Thus, for all that we know, such a complex at two points in time might span non-overlapping parts of the thalamo-cortical system (according to IIT). Imagine that this is true when switching between tasks—say, between watching a movie, which is thought to activate mainly occipital regions if one is immersed in viewing (Aalto et al., 2002; Goldberg et al., 2006), and discussing it, which presumably activates frontal and temporal regions. This would render personal identity virtual even for the shortest of spans; it would also be incompatible with our experience of carrying out prolonged mental operations consciously. Moreover, if my brain at time *t* and time *t* + 1 gives rise to non-overlapping complexes, and so does yours, are those four distinct bursts of experience, or perhaps could one's mind (whatever that means here) transmigrate, given that, as we just noted, IIT cannot tell us anything about conceptual coherence of complexes? Presumably one could argue that the excluded bits of body and brain operating in zombie mode somehow provide this consistency, and anchor such snippets of experience to support the illusion of phenomenal identity and of consistent information processing across time. But given that IIT argues that the only thing that makes a system one is being in an activation state that supports a complex that is completely encapsulated, this account seems problematic^19^.

### Consistency across realizations

ICI computation presupposes that physical states can be parsed into discrete quantities. However, we have no understanding or intuition as to how this choice affects the nature of the resulting complexes, if any, for a given system (a set of transition rules). For all we know, two different choices of number of possible states for each element, together with the selection of the required threshold(s) leads to the emergence of a radically different state of affairs: say, the realization of four equal complexes in one scenario at one spatio-temporal grain, as compared to a single complex at a different grain of the same ICI level. The plausible way out, it seems to us, would be to show that a given architecture gives rise to maximal ICI across all states of activation when described in a particular way in terms of number of states and thresholds. Anything less would render IIT's claims for intrinsic existence unconvincing.

Furthermore, IIT's stipulation that only the maximal ICI exists is potentially problematic in a related sense, given its disregard of inherent constraints on inputs and initial conditions. To compute ICI, one has to explore all logically possible states to determine which states can lead to (from) the current state. However, without ruling out inadmissible inputs and internal states, and without weighting states properly according to the pertinent statistics (say, of possible inputs), the resulting information score could change noticeably. IIT claims that this uniform prior (in Bayesian terms) is intrinsic; however, given that neural networks arguably implement Bayesian computations universally (Knill and Pouget, 2004) and that inadmissible states are ruled out by the laws of physics along with the structure of the mechanisms at work, this reasoning seem less than conclusive. Furthermore, the bipartitions in computing ICI are created by retaining unidirectional connections, rather than by severing connections entirely—a move that could change the ICI values. This design choice is far from obvious; taken together with the two earlier observations, it suggests that IIT needs to naturalize its treatment of normal conditions for it to make a stronger case for indeed being intrinsic.

These question marks have to do to a large extent with computational tractability problem that ICI faces: it can at present be numerically estimated for systems of no more than about 12 binary elements (and the larger the number of supposed states the bigger the problem). Hence, given the extreme computational complexity in estimating Φ, the fundamental properties of this measure remain unclear. Accordingly, the way forward may be for the definitions of the various information measures incorporated into IIT to be extended to continuous measures (Barrett and Seth, 2011), which would allow applying analysis methods to try and systematically (no pun intended) answer such questions. And to reiterate, as to address the issues at hand—demonstrating consistency under exclusion for a given architecture, pinning down the number of emergent minds, determining their “grain” in space and time, ensuring their continuity in space and time, as well as guaranteeing the congruence of the conceptual/perceptual domains in which the realized experiences live—necessitates sweeping across activity states—our notion of dynamic properties appears unavoidable^20^.

To summarize, in its current state of development, IIT's notion of a systemic property leans too heavily toward transient states of activation. Thus, in the terminology of Sections Model Selection, The Boundary Problem, and Inter-Theory Convergence and The Boundary Problem and Systemic Properties, Φ seems to fall somewhere between a systemic property and hot spot analysis.

## The geometric theory revisited

The Geometric Theory of consciousness (GT; Shepard, 1987; Edelman, 1998, 1999; Gärdenfors, 2004; Fekete et al., 2009; Fekete, 2010; Fekete and Edelman, 2011, 2012), like any other computational approach, seeks to understand the formal and structural properties realized by the physical activity of systems that cause consciousness to emerge. Because experience unfolds in time, the basic unit of analysis is taken to be trajectories of (open) dynamical systems. The question thus becomes: what in the composition of such a trajectory makes it more or less fit for realizing phenomenal content?

According to one intuition, it is something about the structure of *this* instance of activity—a given trajectory—that makes it suitable to give rise to *this* bit of phenomenal experience. The GT, however, is founded on the realization that even the simplest phenomenal content (a quale) has relational aspects to it, which implies that it can only exist as a particular manifestation of an entire perceptual/conceptual domain. Thus, *this* activity that gives rise to *this* red is also an example of a category—a region in the space of all hues implemented by the mind in question, which is in turn linked to a multitude of other categories, ultimately making up the phenomenal world, or “reality” as it presents itself to the mind.

This activity is a particular example of another category as well: the category of experiences belonging to a *state* (mode) *of consciousness*. One attribute that differentiates among states of consciousness is the richness of the experienced phenomenal content, which differs across states. Conversely, within a given state of consciousness, the richness (“amount”) of phenomenal content is constant. Because it is the structure of activity trajectories that realizes phenomenal content, it follows that all the trajectories in given a state of consciousness are in some sense structurally equivalent.

Now, as some trajectories do not give rise to phenomenal content at all, by the same logic they must be devoid of such structural features, and complementarily, trajectories associated with increasingly rich experience should exhibit increasingly “rich” structure. Accordingly, the GT posits a notion of complexity that captures the exact structural features corresponding to varying phenomenal richness, that is, to different states of consciousness^21^. By the same token, each state of consciousness—that is, level of complexity of activity—would be associated with a set of all possible activity trajectories that the system could generate when in this state.

The GT is concerned first and foremost with understanding the structure of such trajectory spaces, that is, their geometry. It assumes that trajectory spaces are isomorphic to the perceptual/conceptual domains they realize, which is to say that the formal structure of the physical activity giving rise to consciousness is equivalent to the formal structure of experience^22^ (Shepard, 1987; Edelman, 1998, 1999; Gärdenfors, 2004; Fekete et al., 2009; Fekete, 2010; Fekete and Edelman, 2011, 2012). This implies that every salient feature of experience is expressible in terms of some definite structural feature of a pertinent trajectory space, and, conversely, that salient features of activity trajectory spaces must manifest in experience (if not, why would some structural features of trajectory spaces be associated with phenomenal character while others would not?).

The basic organizational principles of perceptual/conceptual domains are local similarity and topological connectedness: given a segment of experience, arguably there is another possible segment that differs from the first one infinitesimally along any of the dimensions or attributes it consists of, and any such experience can be gradually altered until ultimately a completely different experience emerges. More broadly, the above properties imply that categories—regions in perceptual/conceptual domains—are characterized by family resemblance.

The structure of family resemblance is induced via a distance measure: primal similarity in phenomenal content can be expressed in terms of inverse distance between trajectories in the trajectory space^23^. Just as the members of a category share a family resemblance, the corresponding trajectories are clustered together in the trajectory space. The distance relationships among these clusters are such that they reflect the myriad of similarities and differences between categories (as they are present in experience, not reflected upon). Accordingly, if one were to coarse-grain the space, categories would coalesce to form higher order categories, and so on. In other words, the hierarchical organization of categories expresses itself in a multiscale structure of clusters.

How does the state of consciousness affect this structure? In the absence of consciousness, there should be no such structure; as phenomenality emerges and experience becomes richer, increasingly finer distinctions inhering in the associated phenomenal content become possible. According to the GT, this is brought about by an increasingly elaborate structure of categories reflecting these distinctions. To a first approximation, the complexity of a cluster structure derives from the number of clusters, or rather the number of configurations of clusters (recall that categories themselves can vary continuously, and each configuration can vary in its dimensionality, which loosely corresponds to the number of attributes along which the distinctions differentiating these categories present themselves).

The key constraint on the cluster structures is that they be intrinsic. With this in mind, consider first the extreme case of a geometric object that lacks such structure—for instance, a homogeneous solid volume in space, such as the unit (hyper)cube. Any distinction between regions internal to this volume would necessarily be imposed from the outside and thus not intrinsic. To possess intrinsic structure, the volume cannot be uniformly solid: there must be some “holes” (just like the necessary condition for drawing a shape using only one color is not to cover the entire page uniformly with ink). Accordingly, to detect inherent clustering, one must find such holes, count them, and quantify their dimensionality (cf. a circle has one 1D hole in it, a sphere has one 2D hole, and a torus two 1D holes and a 2D hole; see Appendix 1 in Supplementary Material, Figure S1).

How can holes be detected? Intuitively, by slicing the space in which the object resides into regular cross-sections (for more details see: Fekete et al., 2009; Fekete and Edelman, 2011). While this is an extrinsic measure, which depends on the choice of grain, if the cross-sectioning is carried out across all possible scales, the result is invariant (up to reparametrization). An added benefit is that the resulting descriptor is by definition a multiscale one and thus is sensitive to higher order cluster structure (see Figure S2).

The resulting descriptors are called a multi-scale homology (mSH): for each scale, the result is a vector, holding a hole count for each dimension. For simplicity, let us set aside the scale parameter^24^ and assume that each trajectory space results in a vector that lists the cluster configurations in each dimension, thus expressing the complexity of structure (or the lack thereof) of the respective perceptual/conceptual domain.

Recalling the terminology introduced earlier in this paper, we note that the GT postulates the existence of an MoC: changes in the complexity of activity trajectories go hand in hand with changes in the complexity of the structure of the resulting trajectory space, and thus in the mSH of a (putative) system in a given state of its dynamics (and, if appropriate structure is in place, changes in the state of consciousness). The GT refers to this dual aspect of complexity as *representational capacity*; accordingly, trajectory spaces arising from complex dynamics that exhibit rich intrinsic clustering may be referred to as representational spaces.

We hypothesize that the complexity of trajectory spaces associated with conscious systems is a systemic property, expressed by the mSH associated with a measure of (neural) complexity. That is, in true systems (under this property) the parts interact collectively to produce a conceptual/perceptual space that could not have resulted from the actions of the constituent subsystems in isolation, leading to a richer overall structure of the emergent trajectory space. However, with respect to high dimensional geometry, complexity is not simply additive: merging of low dimensional structure can result in higher level structure, a scenario that would decrease the lower level hole count while increasing the higher level count (see Appendix 2 in Supplementary Material). In other words, according to the GT, intrinsic systems such as (embodied) brains are expected to be alloadditive.

What are the implications of this claim with regard to the system/subsystem problem as it applies to the brain? The various parts of the brain are expected by the GT to interact to produce a trajectory space that is qualitatively different from the spaces produced by any normal cut (and by extension by any subsystem) in that its structure is of greater complexity. As noted, this is likely to manifest as an elaborate higher order structure (that is, an increase in the cluster configuration count in relatively high dimensions), but perhaps this is accompanied by a suppression of low dimensional structure. Perhaps it is best to think of this as a gauge of causal density, as complexity can arise only if there is a tension between local and global dynamics. If the elements of a system are entirely independent, no novel non-trivial global pattern can emerge, whereas if the elements are too strongly coupled, uniform (or at least less structured) dynamics emerges.

The GT thus explains why multi-scale complexity of activity, which brings about non-trivial trajectory spaces and hence nonzero representational capacity, can only arise if the representational labor is divided between elements (units), such that some specialize in (i.e., are more attuned to) lower order features (in terms of geometry) of the representational space (and hence the perceptual/conceptual domain thus realized), whereas others are explicitly tuned to features of varying scope in the categorical hierarchy (Fekete, 2010). This implies that if systems are not coupled in the right manner, their interaction will not promote the emergence of higher order structure, thus failing to form the right kind of (computational) unity necessary to support a systemic property.

Similar considerations apply to the embodiment problem. While at this point the GT is very general and does not offer specific details regarding the nature of the elements that can make up a system that could establish the right kind of dynamics, nevertheless it seems to be more naturally compatible with an outlook in which the major representational heavy lifting carried out by brains is in producing graded electrochemical activity that shifts in time, and not spiking activity (Fekete and Edelman, 2012). If so, it could be argued that many signaling pathways between the brain and the body are similar in kind to the activity produced by the brain, albeit operating on slower time scales. We may then hypothesize that while the body on its own produces a trivial trajectory space, coupling it with a brain profoundly alters the multiscale structure of the trajectory space generated by the brain.

What about hive minds? According to the GT, for minds to fuse and form a collective mind, the emergent joint trajectory space has to be more complex compared to the aggregate of the respective spaces. If we apply the causal density perspective to this scenario, we see that this is an unlikely outcome. Consider the “Chinese gym” scenario (Block, 1980; Searle, 1990). In this scenario, a roomful of non-Chinese speaking men enact a neural network model that converses in Chinese, by each taking the role of a node or synapse. The question of interest is whether understanding of Chinese can thus be achieved (as it clearly cannot by the individuals on their own).

According to the notion of implementation articulated above, the collective in this case seems to merit the “systems reply,” namely, that understanding is to be found at the level of the collective only (Chalmers, 2011). However, according to our analysis (and Chalmers' original one as well), the “gym” does not in fact realize the (combinatorial state) machine (formal structure or algorithm corresponding to the causal organization of the collective) that supposedly understands Chinese as opposed to being able merely to converse in Chinese. This is because in fact each possible transition specified in terms of the single units (the people in the gym) fails to implement the counterfactual stipulations of the form *I*_*i*_⇒*O*_*k*_ (representing an input-output matching, or “strong conditionals” in the language of Chalmers, 1996).

The reason for this is simple: whether or not a person agrees to participate, is paid, or is coerced to do so, the scenario would be far from satisfying such a strong constraint; nothing in the situation will force a person to invariably act stereotypically each and every time across all normal scenarios. Accordingly, one may conclude that the collective structures formed by groups or societies are of a different kind compared to the computational structures realized by brains—both in that they are observer-dependent (and hence extrinsic) and in that they fail to introduce the right kind of causal density necessary to give rise to the requisite non-trivial complexity.

## Discussion

In this paper, we have outlined what we believe are some necessary conditions that an open dynamical system must satisfy if it is to realize phenomenal content. Specifically, properties that we called systemic offer a principled mechanism for singling out intrinsic systems, and thus for banishing the troubling notion of countless redundant/virtual minds formed by subsystems and aggregates of systems, whose existence would otherwise be implied by consciousness being construed as a matter of fact—that is, it being a measurable quantity.

The idea of systemic analysis clearly requires further work. As yet, we cannot offer a concrete example of a system (e.g., a model) that provably possesses the requisite property. In particular, given the lack of understanding of the particulars implied by computational theories of consciousness, it is difficult to establish reasonable normal conditions under which system expansion and contraction can be studied without running the risk of begging the question or missing the point through making arbitrary decisions or letting pre-theoretical commitments creep in unnoticed through this oft-neglected back door.

On a more positive note, the mechanism of system expansion (contraction) constitutes a principled means for understanding the systemic properties of models. For example, through expanding a model to infinity (or contracting it to zero), one can hope to understand if and how certain features of networks (say, patterns of connectivity) systematically increase (decrease) a dynamical property that is thought to promote experience (as in, for instance, the effect of small-world network structure on systemic integrated information).

### Hive minds revisited: Is the US conscious?

An interesting test case for the proposed theory is Schwitzgebel's (2015) recent treatment of the hive mind problem. If consciousness is brought about by complex interactions among a multitude of units, we may be nearing the point at which the interactions among the people in the US would be more complex than those realized by a single brain, from which we may expect the USA to be conscious. According to the analysis offered here, the US is simply an aggregate of systems, such that the trajectory space they jointly realize is simply the “sum” of the individual structures. This is due to the fact that under normal conditions an embodied brain gives rise to the exact same trajectory space if taken as an individual or as part of a collective. We argue that this is because the causal density of interpersonal interactions does not approach that of neurons (and glia) in an embodied brain. However, this is not to deny altogether the possibility of “brain fusion,” which can perhaps be achieved by actually “wiring” embodied brains together (in the sense of setting in place the necessary dense causal structure).

A thought experiment adapted from Schwitzgebel (2015) seems to challenge our notion of systemic properties: if such causally dense connections are formed gradually, we would appear to go from a state in which in an instant the collective minds of the US are wiped out and superseded by a supermind^25^. If we assume that a supermind comes into being, according to our analysis this would mean that the collective gives rise to a trajectory space—and hence a perceptual/conceptual space—that is different from the aggregate of the those of the individuals, and that the underlying dynamics in each and every individual would be different, that is, could only be achieved in the presence of the whole. Therefore, we think that in the process suggested by Schwitzgebel, the individual constituent minds would gradually lose their independence, and as a result the individual minds would be transformed and “phased out,” to be superseded by the supermind. As in any phase transition—that is, switching to a qualitatively different dynamical regime as a result of change in the parameters of the model (in this case the connectivity between units)—this change can be more or less abrupt, and strange things can happen at the transition.

### Systemic properties and the unity of consciousness

Our notion of systemic properties ipso facto affords a naturalistic definition of the unity of experience^26^. This is true both from the perspective of the GT and of IIT, inasmuch as they can be realized in full as research programs. This is because the trajectories of an intrinsic system are by definition unified wholes, that by virtue of the categorical role they play (in the pertinent perceptual/conceptual space) nevertheless exhibit (realize) finer detail. To reiterate, from this perspective unified experiences are the fundamental entities: they can only come into existence through unity (also with regard to the entire possible space of experiences, as that is entailed by realizing a dynamical property).

It is important to note however, that while our framework naturally accommodates the unity of experience, it is agnostic as far as the *experience of unity* goes (Welshon, 2013; Bennett and Hill, 2015; van Leeuwen, 2015): many facets of the content of our experience are “unified” in one sense or another, whether in objects, across different sensory modalities, or in seemingly more fundamental ways such as perspectivalness and even selfhood. However, as both brain-damaged patients and the effects of psychoactive drugs demonstrate (Shanon, 2002; Welshon, 2013; Bennett and Hill, 2015; van Leeuwen, 2015), such variants of content unity likely arise from specific mechanisms and do not constitute necessary aspects of experience, whereas unity in its barest form is simply synonymous with there being something it is like to undergo it.

### Systemic properties and panpsychism

While our analysis rules out naive panpsychist accounts (i.e., that every object has a mind), it does lead one to expect that the prevalence of consciousness in various substrates depends on the MoC that establishes systemic dynamic properties. At this point, it is unclear what the “lower bounds” are in terms of systems that can instantiate the right kind of dynamics. For example, could consciousness arise in organisms without a nervous system? In single cell organisms? In plants? In molecules? This raises another related question: how to construe a null outcome indicated by an MoC. At least in some cases—say, at the limit of contraction of a systemic property, or where an unconscious brain is concerned—one is tempted to invoke the notion of “proto-consciousness,” that is, some minimal glimmer of experience, so far removed from our own that very little can be said of it. Therefore, it would seem that what Chalmers refers to as constitutive panpsychism (or proto panpsychism; Chalmers, 2016) is perhaps compatible with the framework of systemic properties. Be that as it may, our approach implies that such considerations are almost invariably premature before the respective theory is articulated in enough detail, not only formally, but practically (that is, through detailed considerations of implementation).

### Systemic properties and the dynamic nature of consciousness

The GT and IIT differ in many regards, not the least of which is their treatment of the temporal nature of experience. While IIT treats “moments” of experience as discrete episodes, and hence is concerned with mechanisms (systems of interacting elements) in a state (of activation), GT recognizes that computation, and therefore also experience, takes time to unfold. Using considerations of representational capacity, GT shows that the apparent continuity of unfolding experience necessitates time-continuous dynamics, thus concluding that consciousness is in fact dynamic (Fekete and Edelman, 2012).

Nevertheless, even though the dynamic nature of experience remains a polarizing topic both in cognitive science (Herzog et al., 2016) and philosophy (Dainton, 2014), our definition of dynamical properties does not assume that the physical analogs of experience are dynamic, that is, that they are necessarily defined on trajectories and not instantaneous states. This point of contention is therefore beyond the scope of the present discussion.

### Noise, complex dynamics, and implementation

The notion of implementation in our analysis, as mentioned above, presupposes deterministic state transitions in the following sense: that the total state of the system fully determines the next step in a dynamics. Without this stipulation, alluding to the possibility of an intrinsic description, even in an idealized sense, may be seen as unconvincing. However, the current understanding of brain dynamics, and of complex systems in general, suggests that models that do not incorporate stochastic factors cannot quite deal with real life situations satisfactorily—an observation that is seemingly at odds with the stress we place on detail-oriented analysis. This is only an apparent lacuna, as our analysis strictly concerns open systems—systems that are subject at all times to external perturbation. While we referred to the latter as input, a term suggestive of transduction of highly specific environmental events, the framework we describe admits a much wider scope of external influence. Specifically, assuming that a dynamics is fully specified by a system of equations, each equation naturally accommodates both noise and input terms (e.g., as in stochastic differential equations).

It could be argued, that a dynamics driven by noise could hardly produce a stable enough trajectory space, let alone the complex multiscale structure necessary to give rise to phenomenal content. We wholeheartedly agree: this reinforces the idea that for dynamics to support experience there are many hurdles to be cleared. It is for this reason exactly that our nervous system balances between being amenable to external influence and maintaining rich spontaneous (i.e., self-determined) dynamics, as evidenced, amongst other things, by the fact that most of the brain's energy expenditure is directed toward this intrinsic or “resting” activity (Raichle, 2006).

### Concluding remarks

Our analysis offers a naturalistic means of establishing the existence of systems: wholes that are unified dynamically. As such, it can help to dispel concerns arising from various boundary problems that stem from the notion of consciousness being a measurable quantity. As noted earlier, much of its promise is predicated on future development, or rather coming into fruition, of computational approaches to studying experience, such as the Integrated Information Theory or the Geometric Theory of consciousness. Even so, discussing prospects for a solution adds to our conviction that some day it will be possible to understand consciousness fully in a perfectly naturalistic framework, no more and no less so than any other fundamental phenomenon.

## Author contributions

All authors listed, have made substantial, direct and intellectual contribution to the work, and approved it for publication.

### Conflict of interest statement

The authors declare that the research was conducted in the absence of any commercial or financial relationships that could be construed as a potential conflict of interest.

## Footnotes

^1^For this reason, in the present paper we refrain from taking up many of the theoretical distinctions that can be found in the literature, such as the one between “intransitive” and “transitive” consciousness—a distinction that is not made by either of the two theories that we address in depth, the Integrated Information Theory (Oizumi et al., 2014) and the Geometric Theory (Fekete and Edelman, 2011); see Sections Tononi's IIT: Is Integrated Conceptual Information A Systemic Property? and The Geometric Theory Revisited.

^2^This is not to be confused with the experience of unity (Welshon, 2013; van Leeuwen, 2015; and see Section Discussion).

^3^This is to say that the total state of the system at a given time uniquely determines its next state *T*(*t*) ⇒ *T*(*t* + △*t*) (representing an input-output matching, or “strong conditionals” in the language of Chalmers, 1996), or that the conjunction of internal state (and parameters) and input uniquely and invariably determines the subsequent internal state.

^4^Note that both “inputs” in the classical sense, that is, energy transduced by receptors, as well as various types of noise, fall under this description: both can be expressed as terms in the system equations shaping the system's future state. In the brain, in particular, the former sense seems to apply only to a small fraction of the cells, while the latter seems to be prevalent at all levels. In the final section, we will return to the implication of this fact for our notion of implementation.

^5^In systems with time-continuous dynamics, this is a closed interval rather than an ordered set of points in the high-dimensional state space.

^6^Of course, the question of how different is different enough is nontrivial. Below we suggest that a critical determinant in establishing “sameness” in modeling experience is the realization of an intrinsic structure of clusters, something that is a multi-scale invariant of trajectory spaces.

^7^This is crucial not only for effective learning strategies; as vision, for instance, teaches us, brains can only manage to generate meaningful virtual realities and affordances on the fly via bridging assumptions about the statistical nature of the world. Note that this is a computational necessity and not simply a result of the “messy” or limited nature of brains (Wolpert and Macready, 1997).

^8^In the presence of inadmissible inputs or parameter values, a given set of equations may no longer apply to a given physical substrate. Examples include extremely loud sounds that destroy the ear, abnormal concentrations of essential substances like nutrients that can abolish function or even kill biological tissue (and hence systems), high temperature that reduces matter to a plasma, and high mass concentration that results in a singularity.

^9^Whole-part relationships are normally the subject matter of mereology. However, the notion of part that we are developing here places the present discussion largely outside the scope of that discipline.

^10^It is possible that in doing this we gloss over a fundamental feature of brain dynamics, namely, that parameters themselves change, albeit over longer time scales.

^11^Θ denotes the set of all possible parameter values specified by the normal conditions, whereas θ ∈Θ denotes a specific choice of parameters.

^12^A dynamics uniquely determines a trajectory space. The converse is not true strictly speaking; however, up to a relevant notion of equivalence (e.g., diffeomorphism) it is, and moreover, it is hard to see why one would be interested in properties of the model that are not invariant with respect to mere representation. Accordingly, assuming complete congruence seems perfectly sensible.

^13^If *V* is a subset of *U*, the inclusion map is a function i: *U* → *V* that sends every *x* ∈ *V* to *x* ∈ *U* such that *i*(*x*) = *x*. In our case if we consider trajectories of length *M*, and if (*S*, θ) allows for instantaneous states in ℝ^*N*^ (*s*, θ) in ℝ^*k*^ and $\left(\bar{s},\theta \right)$ in ℝ^*N*−*k*^ we define: $s{+}_{i}\bar{s}\equiv i\left(\left(s,\theta \right)\times \left(\bar{s},\theta \right)\right)$ where, $\left(s,\theta \right)\subseteq R^{M\times K},\left(\bar{s},\theta \right)\subseteq R^{M\times \left(N-K\right)},$ × is the Cartesian product, and *i* is the inclusion map. Practically speaking, when comparing simulations of a complete system, and those of a normal cut, this corresponds to attaching to each trajectory in *s* one in $\bar{s}$ when the contracted dynamics are run in parallel (i.e., running the simulation with the pertinent interaction terms set to 0 and evaluating the resulting trajectories as before).

^14^It is unclear at this point—and this may even be a metaphysical question—whether there exists a sufficient level of detail for brain modeling. In practice, there is the constraint of information saturation: once a certain level is reached, limited sensitivity of measurement or computational limitations may preclude further meaningful articulation of the model. This would signify that a “sufficient level of detail” has been attained.

^15^Normal cuts that do not involve single bees would be more difficult to realize, requiring something along the lines of genetic manipulation to create a population of bees with an alternative system of pheromones in our example.

^16^In earlier versions of IIT, the distance was defined as the Kullback–Leibler (KL) divergence, leading to the mutual information as defined in Shannon's information theory. At present, however, IIT is formulated using an actual metric (the KL divergence in not symmetrical) which makes the second I in IIT somewhat metaphorical.

^17^That is, dividing the units into two non-overlapping sets (a normal cut) A and B and computing the II for each of the concepts first with the connections between A and B severed, and then the other way around.

^18^In fact IIT makes a stronger claim: that no overlapping complexes can exist. This is consistent with Tononi's view that only the thalamo-cortical system is responsible for experience. In that case two minds could exist in the split brain syndrome, as the overlap in the systems is outside the “dynamic core” that gives rise to ICI.

^19^In contrast, and account assuming that an embodied brain is necessary for experience would avoid such pitfalls.

^20^In fact the requirements for consistency are more stringent, as we would seek it not only within systems, but across systems: learning, development, and evolution can be viewed as following paths in system space (Yoshimi, 2010). And as the changes along these paths seem for the most part graded and systematic, we would want to know that this is the case for a purported systemic property.

^21^It stands to reason to first develop a measure of neural complexity as pertains to changes in conscious state, mainly in the context of the mammalian brain, as a first step in understanding complexity in brains in general, as well as in aliens or artificial systems. For the purposes of the present discussion, we assume that complexity is a scalar real-valued function. However, there are good reasons to think that this measure should be at least two-dimensional (e.g., to encode qualitative difference as well as “quantity” of richness or level of consciousness; Hobson et al., 2000). This, however, has no direct bearing on the subject matter, as long as such a measure enables readout of level of consciousness, possibly after an additional transformation.

^22^And of course the goal of the GT is to identify such properties. GT argues that this isomorphism should be at least an isometry.

^23^This distance derives naturally from the topological and geometrical attributes of the trajectory space.

^24^The multiscale homology descriptors can be greatly simplified by resorting to persistent homology, resulting in discrete descriptors called barcodes, over which a metric can be defined (Carlsson et al., 2004).

^25^According to IIT one mind would exclude the other at any given point in time, thus predicting a sharp transition from the aggregate to the uber-mind (if the premise is accepted—as noted earlier IIT denies the possibility of this scenario).

^26^We take unity to be “phenomenal unity,” construed as there being something it is like for a subject to undergo an experience that is multifaceted (Bayne and Chalmers, 2003).
