# Supplementary material for: System, Subsystem, Hive: Boundary Problems in Computational Theories of Consciousness
Source: Front Psychol. 2016 Jul 27;7:1041. doi: 10.3389/fpsyg.2016.01041 (PMC4961712; doi:10.3389/fpsyg.2016.01041)
Supplement: Supplementary file 1 [file Appendix1.pdf]

Appendix 1: the dimensionality of holes

In topology, shapes are equivalent up to stretching and deforming, as long as points are not "torn apart", or "glued" together. The simplest topological shape is a (solid) cube. A cube has no holes in it. However, if we puncture it with a needle so that it exits on the other side, we create a hole: to close it we would have to glue points together. Of what dimension is this hole? We can first expand the hole, and at the same time flatten the cube as much as we like, and finally round the corners until we are left with a hoop, and in the realm of pure math, we could continue doing so until we are left with a circle. Around such holes you could place a piece of "string" and fuse its ends together. Once this is done, it could not be contracted to a point while staying on the circle. The hole in a circle is accordingly defined as a 1D hole, as it is demarcated by a 1D manifold (the sting, or rather a closed curve).

A sphere has a hole in it as well, namely, the cavity in the middle. What dimension is this hole? It is not a 1D hole. To see that, imagine drawing a line on the surface of the sphere, say tracing the equator all along its length. This circle however can be smoothly contracted to a point while staying on the sphere (think about gradually shrinking the curve around the equator while moving in towards a pole until they coincide) . What we could do, though, is wrap the sphere with a "sheet of paper" and glue its edges (a closed surface, or a 2D manifold). While staying on the sphere our sheet could not be contracted to a point without ripping, indicating the presence of a 2D hole.

We conclude with a somewhat more complex example: a torus. A torus, like a sphere, has a single 2D hole, which again we see if we wrap it with a sheet, gluing the edges; once this is done, it could not be removed from the torus without tearing it. It also has 2 1D holes: you can

trace two distinct closed curves around the torus's major and minor axes (marked in yellow and red in figure S1): each cannot be shrunk to a point without ripping (as the grid in the figure helps see), and at the same, one ring cannot be smoothly deformed into the other.

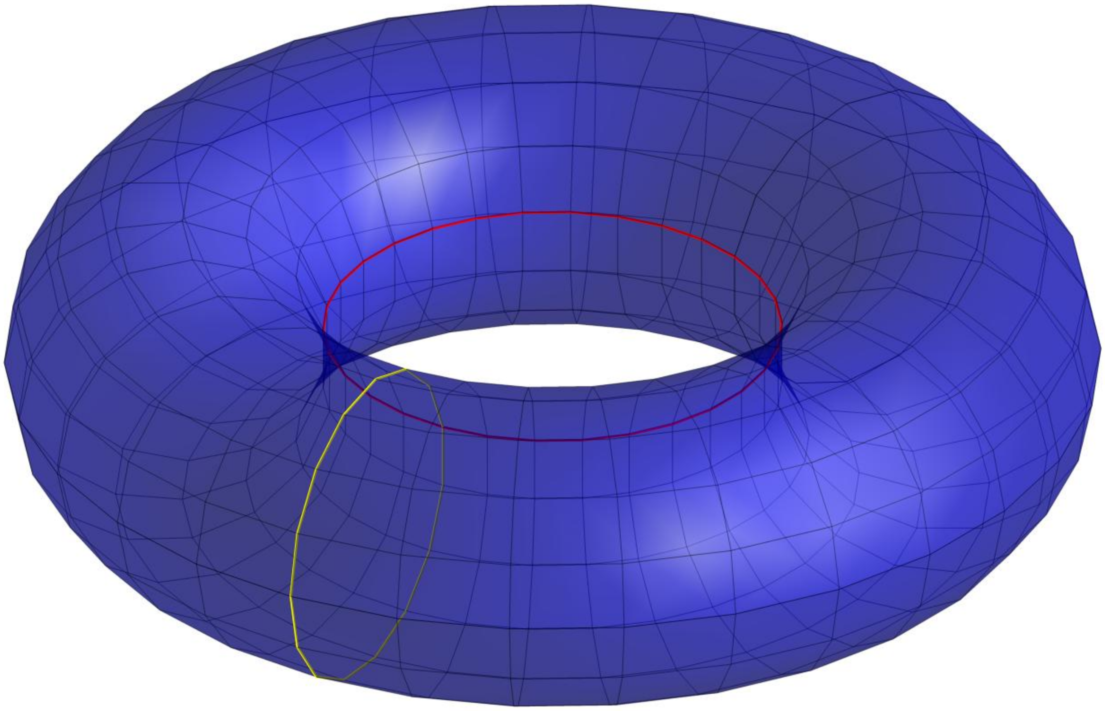

Figure S1: The homology of a torus*. The torus has two 1D holes, highlighted in yellow and red, and a 2D hole, which is demarcated by the blue surface (the surface of the torus).*

The notion of holes generalizes to higher dimensions; in addition, there are also 0-dimensional holes, which are simply the gaps between connected components, although it is probably more intuitive to think of them as simply counting the number of connected components. Therefore a collection of *n* points would have *n* 0-D holes.
