# Supplementary material for: System, Subsystem, Hive: Boundary Problems in Computational Theories of Consciousness
Source: Front Psychol. 2016 Jul 27;7:1041. doi: 10.3389/fpsyg.2016.01041 (PMC4961712; doi:10.3389/fpsyg.2016.01041)
Supplement: Supplementary file 2 [file Appendix2.pdf]

APPENDIX 2: Systemic properties

EXAMPLE 1: The purpose of this example is to provide an illustration of the concept of a systemic property. To do so we will consider a dynamical system comprising elements whose interactions are governed by coupling parameters (connectivity), and show how several distinct patterns of connectivity impact the structure (as measured by homology) of the resulting trajectory spaces.

The Kuramoto Model (Kuramoto, 1975) was suggested to describe the synchrony between oscillators. The phase of each oscillator, θ_i, is evolved according to:

$$\dot{\theta}_i = \omega_i + \frac{1}{N}\sum_{j=1}^{N} K_{ij}\sin(\theta_j - \theta_i)$$

where K_{ij} are the coupling parameters, and ω_i the intrinsic frequency of each oscillator.

Two order parameters can then be defined, r the phase coherence, and ψ the average phase:

$$r(t)e^{-i\psi(t)} = \frac{1}{N}\sum_{j=1}^{N} e^{-i\theta_j(t)}$$

We assume for the purposes of our discussion that the phase represents a movement of an element along a circular path given by $[\cos(\theta_i) \quad \sin(\theta_i)]$. For any trajectory length, the resulting trajectory space for each element will be a circle, denoted by S^1, even though the dimensionality of the embedding space will be $2N$ where N is the length of each trajectory (or rather its discrete representation).

If we are to take a set of uncoupled such elements (i.e. $K_{ij} = 0$) with the appropriate intrinsic frequencies (IFs),[1] the Betti numbers of the resulting trajectory space would be those of an n-torus, because: 1) the movements are independent; 2) therefore the topology of the resulting trajectory space is the Cartesian product of n circles, $\underbrace{S^1 \times \dots \times S^1}_{n}$, which is exactly that of the n-torus; 3) the Betti numbers of the n-torus are given by the coefficients of $(1 + x)^n$.

Elements coupled with a sufficiently large (uniform) K would synchronize perfectly and move in a uniform frequency, therefore regardless of their number their trajectory space would be a circle, that is have the Betti numbers of S^1.

Assume a scenario in which we have three elements, A, B, and C, where elements A and B are symmetrically coupled with $K \gg 1$, and neither is coupled to C. Under this dynamics the Betti numbers of the system AB are subadditive (ignoring the zero Betti number counting connected components, which is trivial for our purposes):

AB has a single normal cut (bipartition) - A/B - which is obtained by evaluating the dynamics on A and B in parallel while the interaction terms between them are set to 0 - $K_{12} = K_{21} = 0$. The normal cut of the system therefore will give rise to a (*2*-)torus trajectory space (a product of two independent circles associated with each of the uncoupled oscillators). Thus, according to the equation above we end with the Betti vector $[1 \quad 2 \quad 1]$. In contrast, as the coupled pair move along a circular path in synchrony, the resulting trajectory space will be circular as well, which will give rise to the Betti vector associated with S^1, $[1 \quad 1 \quad 0]$.

[1] For the uncoupled case that would mean such that no IF is a multiple of any other IF, and for the coupled case, that the IFs of the non coherent elements are not a multiple of the coherent cluster.

Next, if we denote by φ the function that matches a space with its Betti vector we can evaluate it to see if it is systemic. First, we define the relation $>_\varphi$ as:

$$x >_\varphi y \equiv x, y \in R^N \wedge \varphi(x)_1 = \varphi(y)_1 = 1 \wedge \forall_{i>1}\varphi(x)_i > \varphi(y)_i$$

and similarly for $<_\varphi$ where $=_\varphi$ would simply be vector equality. Next we define the relation $+_i$ as: $s{+_i}\bar{s} \equiv i(s \times \bar{s})$ where $(s, \theta) \subseteq R^{M\times L}, (\bar{s}, \theta) \subseteq R^{M\times N-L}$, $\times$ is the Cartesian product, *N* the number of elements in *S*, *L* the number of elements in *s*, *M* the trajectory length and i is the inclusion map. Thus we are left with

$s{+_\varphi}\bar{s} = \varphi(A{+_i}B, K) >_\varphi \varphi(A, K) = S$. Therefore A an B are proper parts of AB, and because for every normal cut φ is larger than when evaluated on the entire system, φ is a subadditive conjoint property.

In contrast, φ is not conjoint for ABC: First, for ABC, because C is unconnected from both A and B, the "entire system" and the normal cut AB/C are one and the same. Therefore both give rise to the same trajectory space, a torus, with the corresponding Betti vector $[1 \quad 2 \quad 1]$. Thus AB and C are not proper parts of ABC, therefore φ cannot be conjoint for ABC. Accordingly φ is systemic for AB - as it is conjoint for AB and AB is not a proper part of ABC.

EXAMPLE 2: In this example, we wish to show another simple system giving rise to a systemic property under a definite pattern of connectivity, which, unlike our previous example, is super-additive.

Consider the following system of second order ODEs, described schematically in figure S3A in which *N* Duffing oscillators (Duffing, 1918), $X_i, i = 1..N$, are unidirectionally connected to form a ring (Perlikowski et al., 2010) :

$\ddot{x}_i = -b\dot{x}_i - ax_i - x_i^3 + k_i(x_{i-1} - x_i)$

which can be represented as the first order set of equations as follows:

$\dot{x}_i = x_{i+N}\ , \dot{x}_{i+N} = -bx_{i+N} - ax_i - x_i^3 + k_i(x_{i-1} - x_i)$

If two such oscillators are coupled, following the above equations, this results in a space of trivial structure, whereas coupling 3 or more elements with an appropriate coupling parameter (e.g. 0.3) will result in a ring structure (figure S3B,C), while each element on its own will simply stabilize at 0.

Consider for example the system $X_1X_2X_3X_4$, arranged on a ring with a coupling parameter of 0.3. The system will therefore give rise to a circular trajectory space with the associated Betti vector $[1 \quad 1 \quad 0]$. A normal cut would result if the connections between the nodes in each of the two groups are set to 0. An example would be X_1X_3/X_2X_4 where $k_1, k_2, k_3\ and\ k_4$ would be set to 0, or $X_1X_2X_3/X_4$ in which $k_3\ and\ k_4$ would be set to 0. However, any normal cut will compromise this ring connectivity pattern, resulting in an unstructured trajectory space - a single point in the first scenario, and an unstructured cloud in the second. Both cases result in the homology of a single connected component - $[1 \quad 0 \quad 0]$, and therefore any part of $X_1X_2X_3X_4$ is a proper part. Thus for this architecture, the homology of the trajectory space would be a super-additive conjoint property, and as is this case it is the complete system, it is not a proper part, and hence systemic.

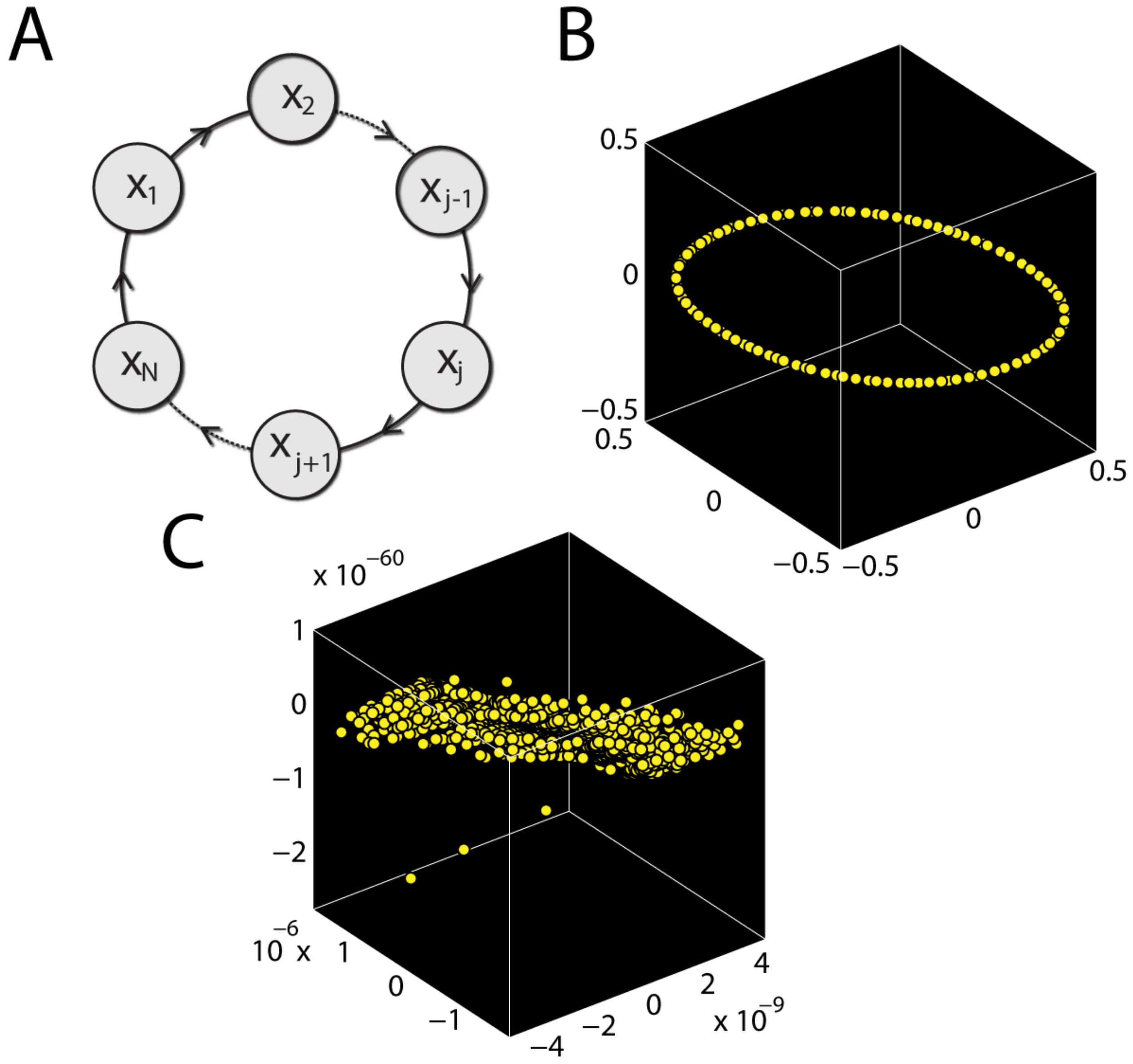

Figure S3: a ring of unidirectionally coupled systems. *(A) the topology (architecture) of the system (B) a point cloud depicting the state variables for a ring of three oscillators with a coupling parameter 0.3. (C) a point cloud depicting the state variables of a ring of two coupled oscillators.*
