# Supplementary material for: System, Subsystem, Hive: Boundary Problems in Computational Theories of Consciousness
Source: Front Psychol. 2016 Jul 27;7:1041. doi: 10.3389/fpsyg.2016.01041 (PMC4961712; doi:10.3389/fpsyg.2016.01041)
Supplement: Supplementary file 3 [file SupplementaryFigure2.pdf]

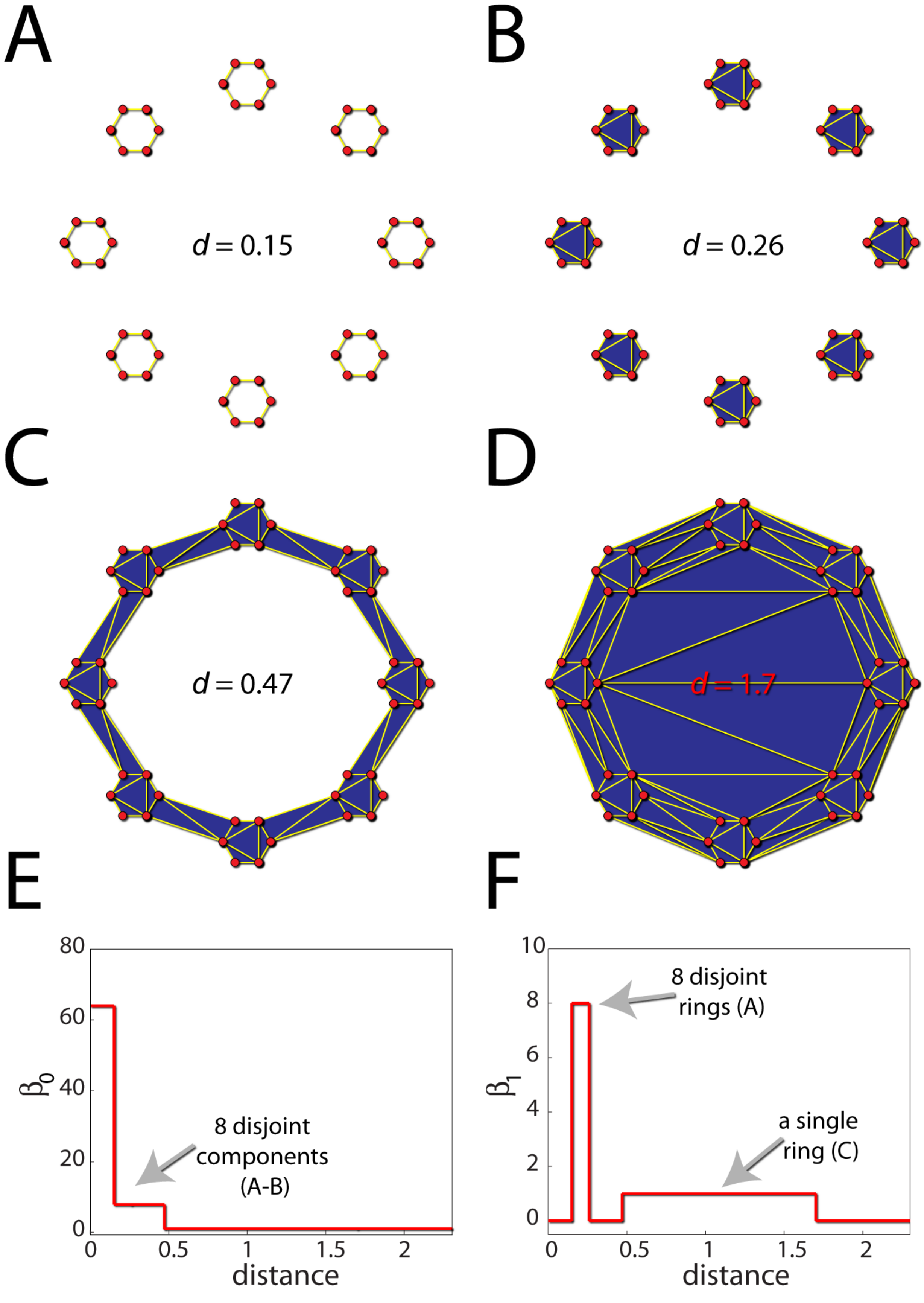
A
d = 0.15
B
d = 0.26
C
d = 0.47
D
d = 1.7
E
80
60
40
20
0
β0
8 disjoint
components
(A-B)
0
0.5
1
1.5
2
distance
F
10
8
6
4
2
0
β1
8 disjoint
rings (A)
a single
ring (C)
0
0.5
1
1.5
2
distance

***Figure S2: Multiscale homology**. 64 points arranged along a circle in 8 circular clusters. After computing the distance between each pair of points a* **simplicial complex** *can be "grown" as a function of scale (distance) by adding edges connecting points for each scale d in case the distance between the points is smaller than d. If three points are connected by edges, the surface (triangle) is added to the complex as well (and so on for higher dimensions). Initially, we start with an isolated vertex (and hence a connected component) for each point. (A) Increasing the maximal allowed point to point distance, the first edges are added. As the points are equally spaced on the smaller circles, this causes the emergence of eight open circles, which are associated with 8 1-D holes. At the same time the number of connected components decreases to 8. (B) As the scale is increased, more edges add to the complex, and with them the corresponding surfaces (faces). The complex now still has 8 connected components, but no longer holes of higher dimensions. (C) As the scale is increased further, the eight clusters connect. Now there is a single connected component in the complex, and a single 1-D hole resulting from the larger circle. (D) Finally, the complex completes, the ring structure no longer exists, only a simply connected convex shape. (E) 0-D holes (counting connected components) as a function of scale. The graph reflects the initial point cloud: 64 points in total, the joining of each cluster, followed by the entire cloud joining. (F) 1-D holes as a function of scale. As can be seen there are two non-zero scale intervals in which 2D structure manifests. Note that the larger scale feature persists for a longer interval. This is typical of multiscale structure, which at times can also exhibit scale-free behavior that manifests itself in a power law of persistence intervals.*
